# Recent advances in stereoselective 1,2-*cis*-*O*-glycosylations

**DOI:** 10.3389/fchem.2022.972429

**Published:** 2022-08-19

**Authors:** Akihiro Ishiwata, Katsunori Tanaka, Jiaming Ao, Feiqing Ding, Yukishige Ito

**Affiliations:** ^1^ RIKEN Cluster for Pioneering Research, Saitama, Japan; ^2^ School of Materials and Chemical Technology, Tokyo Institute of Technology, Tokyo, Japan; ^3^ School of Pharmaceutical Sciences (Shenzhen), Sun Yat-Sen University, Shenzhen, China; ^4^ Graduate School of Science, Osaka University, Osaka, Japan

**Keywords:** 1,2-*cis* glycosylation, stereoselective assembly, method for activation of glycosylation, bimodal glycosylation, intramolecular aglycon delivery (IAD)

## Abstract

For the stereoselective assembly of bioactive glycans with various functions, 1,2-*cis*-*O*-glycosylation is one of the most essential issues in synthetic carbohydrate chemistry. The *cis*-configured *O*-glycosidic linkages to the substituents at two positions of the non-reducing side residue of the glycosides such as α-glucopyranoside, α-galactopyranoside, β-mannopyranoside, β-arabinofuranoside, and other rather rare glycosides are found in natural glycans, including glycoconjugate (glycoproteins, glycolipids, proteoglycans, and microbial polysaccharides) and glycoside natural products. The way to 1,2-*trans* isomers is well sophisticated by using the effect of neighboring group participation from the most effective and kinetically favored C-2 substituent such as an acyl group, although high stereoselective synthesis of 1,2-*cis* glycosides without formation of 1,2-*trans* isomers is far less straightforward. Although the key factors that control the stereoselectivity of glycosylation are largely understood since chemical glycosylation was considered to be one of the useful methods to obtain glycosidic linkages as the alternative way of isolation from natural sources, strictly controlled formation of these 1,2-*cis* glycosides is generally difficult. This minireview introduces some of the recent advances in the development of 1,2-*cis* selective glycosylations, including the quite recent developments in glycosyl donor modification, reaction conditions, and methods for activation of intermolecular glycosylation, including the bimodal glycosylation strategy for 1,2-*cis* and 1,2-*trans* glycosides, as well as intramolecular glycosylations, including recent applications of NAP-ether-mediated intramolecular aglycon delivery.

## Introduction

**FIGURE 1:**
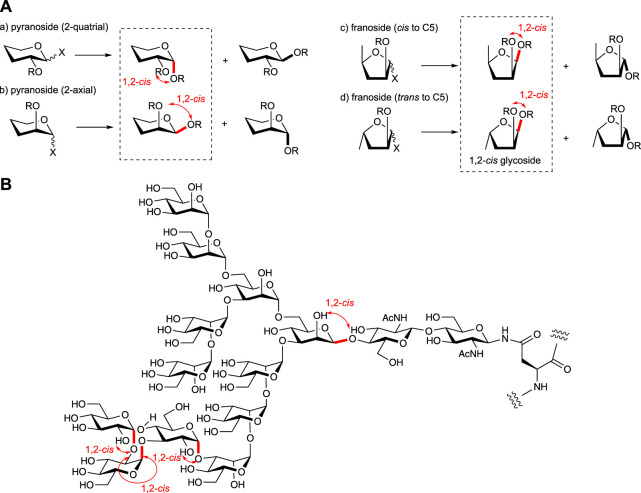
**(A)** Various types of glycosylations and **(B)** 1,2-*cis* glycosides in nature (high mannose-type *N*-glycan is shown as the example).

Stereoselective *O*-glycosylation is essential for achieving the facile assembly of biologically relevant oligosaccharides (Figure 1A). The *cis*-configured *O*-glycosidic linkages to the substituents at two positions of the non-reducing side residue of the glycosides such as α-glucopyranoside, α-galactopyranoside, β-mannopyranoside, β-arabinofuranoside, and other rather rare glycosides are found in natural glycans including glycoconjugates (glycoproteins, glycolipids, proteoglycans, and microbial polysaccharides) and glycoside natural products (Figure 1B). The way to 1,2-*trans* isomers is well sophisticated due to the effect of neighboring group participation from the most effective and kinetically favored C-2 substituent such as an acyl group, although high stereoselective synthesis of 1,2-*cis* glycosides without formation of the 1,2-*trans* isomer is far less straightforward. Although the key factors that control the stereoselectivity of glycosylation are largely understood since chemical glycosylation was considered to be one of the useful methods to obtain glycosidic linkages as an alternative way of isolation from natural sources, controlling the stereoselectivity of the formation of 1,2-*cis* glycoside is extremely challenging in synthetic chemistry, as in the case of α-gluco (2-equatrial)- and β-manno (2-axial)-type glycoside formations (Figures 1Aa,b). To overcome this problem, various methods have been developed for the stereoselective synthesis of more difficult equatorial glycosides such as β-mannoside found in the core structure of the *N*-glycans (recent review; Ding et al., 2022a). This mini review enclosed the recent advances in stereoselective 1,2-*cis*-*O*-glycosylation (recent reviews; Nigudkar and Demchenko, 2015; Takahashi and Toshima, 2021; Manabe, 2021; Mukherjee et al., 2022) for the synthesis of various naturally occurring glycan structures. Donor structures are mainly focused on versatile glycosylation with various acceptor molecules (recent review; Leng et al., 2018) although the acceptor reactivity is also well-known as the important factor in controlling the selectivity of glycosylations (recent review; van der Vorm et al., 2019).

## Recent advances on 1,2-*cis* glycosylations by intermolecular coupling

### Recent development based on glycosyl donor modifications and reaction conditions for 1,2-*cis* glycosylation

**FIGURE 2:**
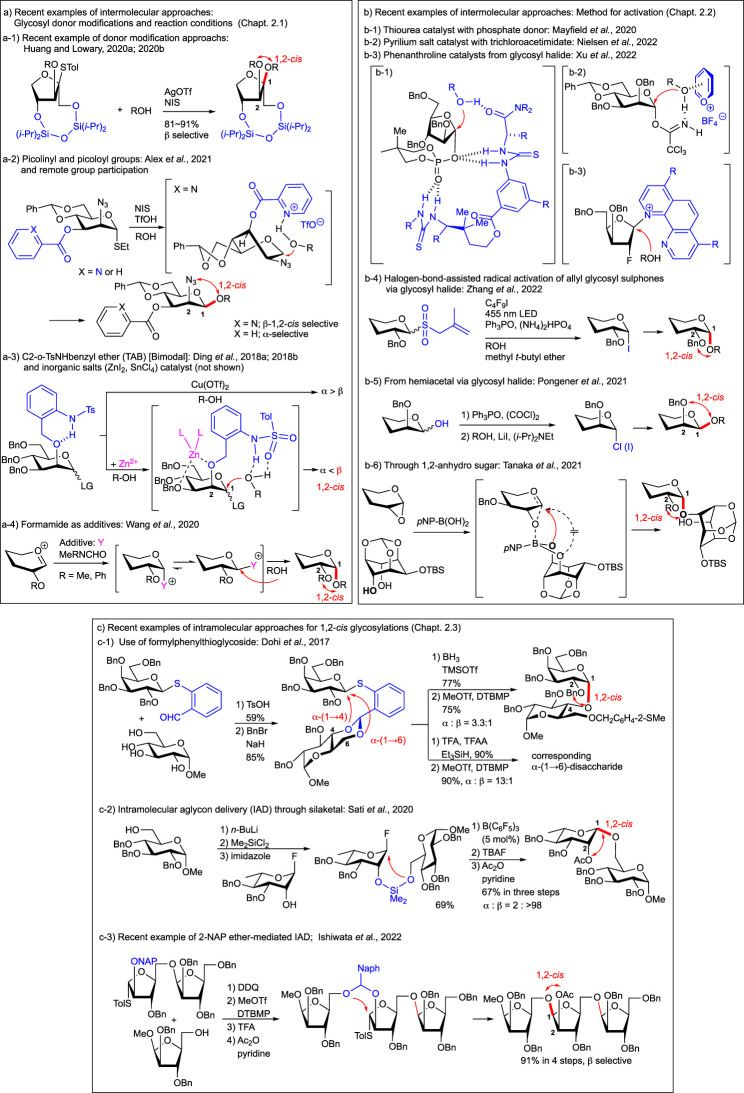
Recent advances in 1,2-cis glycosylations: some examples. **(A)** Recent examples of intermolecular approaches: Glycosyl donor modifications and reaction conditions. a-1) Recent example of donor modification approachs; a-2) Picolinyl and picoloyl groups and remote group participation; a-3) C2-o-TsNHbenzyl ether (TAB) [Bimodal] and inorganic salts (ZnI_2_, SnCl_4_) catalyst (not shown); a-4) Formamide as additives. **(B)** Recent examples of intermolecular approaches: Method for activation. b-1) Thiourea catalyst with phosphate donor; b-2) Pyrilium salt catalyst with trichloroacetimidate; b-3) Phenanthroline catalysts from glycosyl halide; b-4) halogen-bond-assisted radical activation of allyl glycosyl sulphones via glycosyl halide: b-5) From hemiacetal *via* glycosyl halide; b-6) Through 1,2-anhydro sugar. **(C)** Recent examples of intramolecular approaches for 1,2-cis glycosylations. c-1) Use of formylphenylthioglycoside; c-2) Intramolecular aglycon delivery (IAD) through silaketal; c-3) Recent example of 2-NAP ether-mediated IAD.

For controlling the stereoselectivity of glycosylation, the protective groups on the donor moiety are well studied as one of the main factors (recent review, Csávás et al., 2021), including chiral auxiliary at 2-position (recent review, Mensink and Boltje, 2017). The cyclic protective groups on diols for the conformationally constrained donors (Jeanneret et al., 2020) could also be used for various glycosyl donors as one of the key stereocontrolling factors for glycosylation. The di-*t*-butylsilylene (DTBS) group, which was used to construct 1,2-*cis* glycoside as the protective group of the 4,6-*O*-galactosyl type donor (Imamura et al., 2003; Imamura et al., 2008a) and also used for GalNAc derivatives (Imamura et al., 2008b), was applied to the synthesis of the all-1,2-*cis*-linked repeating unit from the *Acinetobacter baumannii* D78 capsular polysaccharide (Njeri and Ragains, 2022). The cyclic protective groups are also effective in the case of well-studied arabinofuranosylations (review, Imamura and Lowary, 2011) by using 3,5-*O*- (Ishiwata et al., 2006; Zhu et al., 2006; Crich et al., 2007) and 2,3-*O*- (Ishiwata et al., 2006; Imamura and Lowary, 2010) cyclic protective groups such as DTBS, benzylidene, tetraisopropyldisiloxanilidene (TIPDS), and xylylene groups. These protective groups were used for conformational fixation of the flexible five-membered furanoside structure (Figure 1Ac) by the formation of bicyclic fused rings to control the approach of acceptor molecules. As an alternative use of the cyclic protective group on the furanoside ring, the 1,4-*O*-TIPDS-protected xylurofuranosyl donor has been developed for specific glycosylation to obtain 1,2-*cis* glycosides with various acceptors (Figure 2A1) (Huang and Lowary, 2020a; Huang and Lowary, 2020b) as in the case of a similarly constrained fructofuranosyl donor whose protective group is blocking one side of the approach of the acceptor (Oscarson and Sehgelmeble, 2000).

The protective group with hydrogen-bonding property such as picolinyl and picoloyl groups (Figure 2A2) (Pistorio et al., 2014; Alex et al., 2020) acts as the stereo-directing group for 1,2-*cis* glycosylation (Loh, 2021; recent review, Khanam and Mandal, 2022), which can also be applied to selective β-arabinofuranosylation (Li S et al., 2018) and synthesis of natual prodiuct such as Tiacumicin B (Norsikian et al., 2020; Tresse et al., 2021). The direct intramolecular neighboring and remote group participation of these groups to glycosyl cation led to the *trans*-glycosylation of the substituents as well (Yasomanee and Demchenko, 2012; McMillan and Crich, 2022).

Bimodal donors (recent review, Ding et al., 2022b) equipped with C2-*o*-TsNHbenzyl ether (TAB) not only for gluco-type glycosylation but also for manno-type glycosylation can be transformed to both anomers simply by switching reaction conditions (Figure 2A3) (Ding et al., 2018a; Ding et al., 2018b) whose optimizations have been carried out, including on the solvent (Ishiwata et al., 2008a; recent review, Mong et al., 2017) and on the concentrations (some examples: Chao et al., 2009; 2011; Ishiwata et al., 2010a; Kononov et al., 2012) of the *O*-glycosylation. In the case of gluco-type glycosylation of the trichloroacetimidate donor, screening of the reaction conditions revealed that TfOH was the most effective to afford 1,2-*cis* α-selective glycosylation at a lower concentration at room temperature and that triflimide (Tf_2_NH) (Kowalska and Pedersen, 2017) resulted in nearly complete 1,2-*trans* β-selectivity in EtCN at −78°C. On the other hand in the case of manno-type glycosylation, further screening of the catalyst (recent review, Nielsen and Pedersen, 2018) and the leaving group and thermodynamic conditions (Adamo and Kovác, 2007; Hou and Kovác, 2010) revealed that glycosyl diphenylphosphite (Kondo et al., 1994) was the best among all tested and catalytic amounts of Cu(OTf)_2_ (Mukaiyama et al., 1979; Sato et al., 1986) at 80°C or two equivalents of ZnI_2_ at −10°C afforded 1,2-*trans* α- or 1,2-*cis* β-selective glycosylations, respectively. The experimental results suggest that ZnI_2_ breaks the internal hydrogen bonding of the C2-*o*-TsNH benzyl group between C2-O and NH by coordination of one equivalent of ZnI_2_ with ether oxygen at *cis*-configured 2- and 3-positions of the mannosyl donor. The applications of this bimodal methodology to the stereocontrolled assembly of naturally occurring glucans having α/β-linkages to various positions of acceptors and branches have been shown recently (Ding et al., 2020).

As shown in many cases with donor modifications, reagent-controlled glycosylation (Yao et al., 2019) is also an important way for 1,2-*cis* glycosides. Recently, some practical methodologies have been reported. Additives such as DMF (Koto et al., 1984; Sato et al., 1986) and Ph_3_P=O were effectively used for the stereoselective construction of α-glucosyl linkages to secondary alcohols with TMSOTf and primary alcohols with TMSI, respectively (Wang et al., 2018; Njeri et al., 2021). The alternative nucleophilic additive for α-glycosylation methyl (phenyl) formamide (MPF) was found and applied to the synthesis of α-(1,4)-glucosamine and α-(1,4)-galactosamine linkages (Figure 2A4) (Wang et al., 2020; Zhang C et al., 2022). A simple ZnI_2_-directed strategy for 1,2-*cis* glycosylation bearing 4,6-*O*-tethered (Crich and Chandrasekera, 2004) glucosyl and mannosyl trichloroacetimidate donors has been developed with excellent stereoselectivity (Ding et al., 2021; Zhong et al., 2021). This simple strategy by the direction of SnCl_4_ instead of ZnI_2_ at −40°C afforded 1,2-*cis* glycoside when 0.1 equivalent was used, and by using three equivalents of SnCl_4_ at room temperature, we obtained 1,2-*trans* glycoside *via* product isomerization through plausible endo-cleavage supported by DFT calculations (Zhong et al., 2022). This also provides a more simple, mild, and effective bimodal glycosylation method. On the other hand, the recent examples for remote group participation (Hansen et al., 2020; Hettikankanamalage et al., 2020; Upadhyaya et al., 2021) introduced the 1,2-*cis* glycosylations selectively and practically from 6- [2,2-dimethyl-2-(*ortho*-nitrophenyl)acetyl: Liu et al., 2019; benzoyl: Shadrick et al., 2020; -C(=NPh)CF_3_: Liu et al., 2022a], 4- (levulinoyl: Zhang et al., 2021), and 3- [2-(diphenylphosphinoyl) acetyl: Liu et al., 2022a; Liu et al., 2022b] positions.

### Recent advances in the method for activation of 1,2-*cis* glycosylation

In addition to various furanosyl phosphate donors (Figures 1Ac,d, 2B1) (Mayfield et al., 2020), the suitably protected mannosyl and rhamnosyl diphenylphosphate donors have been reported to be activated with the bis-thiourea catalyst as one of the organocatalytic approaches (Bradshaw et al., 2018; Xu and Loh, 2018 recent reviews, Mayfield et al., 2020; Park et al., 2017; ) afforded 1,2-*cis* glycoside in a highly selective manner (Li et al., 2020). It is noteworthy that a Schreiner thiourea catalyst with a halogen bond donor such as 2-iodoimidazolium salt has been developed to afford 1,2-*cis N*-glycoside from glycosyl trichloroacetimidate and amide of protected Asn (Li G et al., 2018). Pyrilium salt as an alternative organocatalyst effectively promotes the glycosylation of α and β-glycosyl trichloroacetimidate *via* S_N_2-type inversion to afford β- (1,2-*cis* manno-) and α- (1,2-*cis* gluco-) glycosides, respectively (Figure 2B2) (Nielsen et al., 2022).

Glycosyl halides are still considered one of the most useful and reactive intermediates for glycosidic bond formation as follows. First of all, promoted by phenanthroline catalysts as a recent alternative organocatalytic approach, various glycosyl bromide glycosides including both pyrano- and furanosides (as well as 2-fluoro sugars ) afforded 1,2-*cis* glycoside through glycosyl phenanthrolinium ion intermediates (Figure 2B3) (Yu et al., 2019; DeMent et al., 2021; Li and Nguyen, 2021; Xu et al., 2022). Second, halogen bond-assisted radical activation of allyl glycosyl sulphones was employed by forming halogen bond (review, Cavallo et al., 2016) complexes with perfluoroalkyl iodides under visible light irradiation conditions (Wan et al., 2021) *via* glycosyl radical intermediates (recent review, Chen et al., 2021) to give glycosyl iodides as the intermediate for glycosylation. Subsequent glycosylation in one pot afforded 1,2-*cis* glycoside by the effect of the ether solvent (Figure 2B4) (Zhang Y et al., 2022). Third, from the hemiacetal of mannose and rhamnose, 1,2-*cis*-β glycosides were obtained *via* dehydrative halogenation, followed by α-iodide formation mediated by lithium iodide (Figure 2B5) (Pongener et al., 2021). Since halide was used as the key intermediate in the latter two cases, the well-studied chemistry of halides for stereoselective glycosylation could be simply applied.

1,2-Anhydro sugar (Halcomb and Danishefsky., 1989; recent review; Li H et al., 2018), one of the activated forms of the 2-hydroxy-hemiacetal as an ultimately participated epoxide structure to give stereoelectronically favored 1,2-*trans* isomer, reacted from the opposite side of oxygen of epoxide, applied recently to regioselective (Tomita et al., 2020) and diastereoselective desymmetric 1,2-*cis* glycoside formations (Figure 2B6) (Tanaka et al., 2020) by the action of tetrahydroxydiboron with *trans*-diol and *p*-nitrophenylboronic acid with *meso*-diol, respectively (recent review, Takahashi et al., 2022). This S_N_i-type approach to 1,2-*cis* products supported by mechanistic studies was applied to the synthesis of core structures of phosphatidylinositolmannosides (PIMs) and glycosylphosphatidylinositol (GPI) anchors, as well as the common β-mannoside structure of the LLBM-782 series of antibiotics from *meso*-diol of *m*-inositol derivatives.

### Recent examples using intramolecular coupling for 1,2-*cis* glycosylation

In order to get the 1,2-*cis* glycoside, stereospecifically, the procedure based on intramolecular aglycon delivery (IAD) (Barresi and Hindsgaul, 1991) (recent reviews, Ishiwata and Ito, 2017; Ishiwata, 2019; Fairbanks, 2021) is still one of the most promising methodologies despite the initial tethering between the glycosyl donor and acceptor residues before the intramolecular glycosidic bond formation reaction. However, a two-step procedure can precisely control the approach of the oxygen atom of the hydroxy group in the acceptor residue which was linked as the mixed acetal to the donor residue. IAD had been applied, especially to the β-mannoside linkage found in the core structure of the *N*-glycans, as one of the most difficult and attractive synthetic targets on stereoselective glycosylation. Although the effort on various intramolecular glycosylations *via* tethering using two non-reacting functional groups of both donors and acceptors has also been carried out for both 1,2-*cis* and 1,2-*trans* selective glycosylations (Jia and Demchenko, 2017), it has been shown as one of the alternative intramolecular methods that the leaving group functionalization in the donor moiety as in the case of *o*-formylphenyl thioglycoside obtained from non-malodurous calicyl-type thioglycoside (Dohi et al., 2021) can be used for the tethering with the diol acceptor and regioselective ring opening of *S*-donor-substituted benzylidene acetal, followed by intramolecular glycosylation with the resultant exposed hydroxy group to afford 1,2-*cis* glycosides over three steps (Figure 2C1) (Dohi et al., 2017). Improvements for IAD have also been achieved by tethering to hydroxy groups on both residues through the silaketal (Stork and Kim, 1992) from sugar silanes (Figure 2C2) (Walk et al., 2015; Sati et al., 2020). In the case of the IAD, the 2-*O*-mixed acetal linkage and the axial *O*-mixed acetal substituent as the precursor for intramolecular transfer seem to be kinetically and stereoelectronically favored as in the case of neighboring group participation of acyl groups. *p*-Methoxybenzyl (PMB) ether-mediated IAD was well known as the most practical method to be applied for the synthesis of β-mannoside in *N*-glycan (Ito and Ogawa, 1994). The effective oxidative one-step linking of axially configured 2-*O*-PMB ether to produce a corresponding *p*-methoxybenzylidene mixed acetal at the 2-*O*-position of the mannosy donor with the acceptor as an aglycone. For this method to be more versatile, suitable stabilization of the mixed acetal intermediate by introducing the 2-naphthylidene acetal (Ishiwata et al., 2008b; reviews, Ishiwata et al., 2010b; Ishiwata and Ito, 2012) has been developed for various 1,2-*cis* linkages for application to the synthesis of plant β-L-arabinofuranosides (Figure 2C3) (Kaeothip et al., 2013a; Kaeothip et al., 2013b; Ishiwata et al., 2014; Ishiwata et al., 2022) and various other types of glycosides (Ishiwata et al., 2011; Ishiwata and Ito, 2011; Tamigney et al., 2014; Robinson et al., 2020) including β-L-rhamnosyl linkage (Lee et al., 2008; Yu et al., 2016; recent review, Rai and Kulkarni, 2021).

## Summary

This minireview introduced some of the recent advances in the development of stereoselective 1,2-*cis*-*O*-glycosylation, for the synthesis of various naturally occurring glycan structures possessing α-glucopyranoside, α-galactopyranoside, β-mannopyranoside, β-arabinofuranoside, and other rare glycosides. Donor structures that mainly focused on versatile glycosylation with various acceptor molecules were shown from recent examples and from further matured ones, such as controlling by glycosyl donor modification and reaction conditions and novel methods for activation of intermolecular glycosylation including the bimodal glycosylation strategy for 1,2-*cis* and 1,2-*trans* glycosides, as well as intramolecular glycosylations, including recent applications of NAP-ether-mediated intramolecular aglycon delivery. As in the case of novel methods for activation through glycosyl halides shown in this minireview, it was strongly suggested that the previously studied chemistry of known glycosyl donors for stereoselective glycosylation, including 1,2-*cis* glycosylation, has similar potential to be simply applied in combination with the novel activation methodology, although it must be well optimized as demonstrated previously for practical use. In many recent cases, the results of stereoselectivity and the pathways of glycosylations have been explained by mechanistic studies using highly optimized density functional theory (DFT) calculations and other organic and enzymatic reactions for our better understanding. In the case of many examples shown in this minireview, hybrid functionals such as B3LYP and M06, as well as double hybrid, ωB97X-D were selected to be used as various basis sets [6-31 + G(d,p), 6-31G(d), 6-31G*, 6-31 + G**, Def2SVPP and Def2TZVP] with/without D3BJ dispersion corrections and in combination with a polarizable continuum model (PCM) or implicit solvation model based on density (SMD) for each solvent. DFT calculations could discuss the evidence of glycosyl cation species as well (review, Merino et al., 2021). However, it is still difficult to compare with other experimental results in different studies as we always had a difficult time understanding the outcome of glycosylation and predicting the reactivity, especially stereoselectivity (Chang et al., 2020; Chang et al., 2021). Only through our continuous effort to gain a clear understanding of it from a multidirectional point of view, 1,2-*cis* glycosylations would be finally controlled to be well-predictable as 1,2-*trans* glycosylation.
